# A cross-sectional study evaluating the prevalence and predictors of malnutrition among children and adolescents visiting an urban academic hospital in Nepal

**DOI:** 10.1017/S136898002300188X

**Published:** 2023-12

**Authors:** Ram Chapagain, Bishnu Giri, Tribhuwan Bhattarai, Juna Dhungana, Michelle Walters, Erika Damasco, Jessica Blanco, Kyriaki D Ladas, Andreas Antoniades, Elena Ladas

**Affiliations:** 1 Department of Paediatrics, Kanti Children’s Hospital, National Academy of Medical Sciences (NAMS), Kathmandu, Nepal; 2 Oncology Unit, Kanti Children’s Hospital, Kathmandu, Nepal; 3 Division of Hematology/Oncology/Stem Cell Transplant, Department of Pediatrics, Columbia University, Irving Medical Centre, 3959 Broadway, CHN 10-06A, New York, NY 10032, USA; 4 International Agency for Research on Cancer, World Health Organization, Lyon, France; 5 Aristotle University School of Medicine, Thessaloniki, Greece

**Keywords:** Hospital malnutrition, Stunting, Nutritional status, Food insecurity, Nepal

## Abstract

**Objective:**

To examine the prevalence of malnutrition among children and adolescents visiting Kanti Children’s Hospital (KCH) and identify predictors associated with malnutrition. Results will guide the development of a newly established nutrition programme at KCH.

**Design:**

This cross-sectional pilot study recruited children and adolescents over a 1-month period. Nutritional anthropometrics (height, weight and mid-upper arm circumference (MUAC)) and socio-demographic questionnaires were administered. Clinical data were abstracted from the medical chart.

**Setting:**

KCH in Kathmandu, Nepal.

**Participants:**

370 children and adolescents.

**Results:**

Most participants were male (65·1 %); mean age was 3·9 years (±3·4 years). The prevalence of stunting was 25·9 %, wasting was 17·3 % and 24·0 % when classified by BMI-for-age *Z*-score or MUAC, respectively. Two percent of participants were overweight. Notably, 32·1 % of children ≥5 years were classified with wasting based on MUAC-for-age *Z*-score, which is higher than that observed in children <5 (20·2 %). Food insecurity was reported among 58·2 % of children with stunting and 34·0 % with wasting. Chronic medical conditions predicted stunting and wasting. The lowest level of wealth predicted stunting, while ethnicity predicted wasting. Ethnicity and education level predicted food insecurity.

**Conclusions:**

We found that the prevalence of stunting and wasting at KCH are higher than previously published studies in Nepal. Malnutrition persists beyond 5 years, and we identified several predictors of malnutrition. Increased provision of and access to clinical nutrition programmes is an essential need for KCH. Twinning programs that provide local clinicians with increased opportunities for education and mentorship of local staff remains a pressing need in Nepal.

## Graphical abstract

**Figure:**
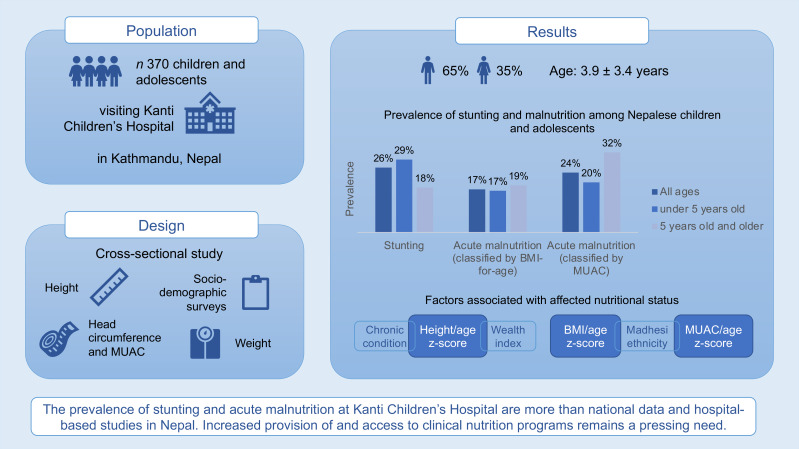

Nepal has been recognised as an international leader in advancing the health of children. Nepalese children are among the highest to be fully vaccinated,^(1)^ and the prevalence of childhood night blindness has decreased from 1·4 % in 1989^(2)^ to 0·27 % in 1998^(3)^ due to the National Vitamin A program that was established in 1993. Progress in the nutritional health of Nepalese children has been largely realised from expanded health coverage, particularly in rural areas where Nepal’s large network of female health volunteers have led community-based programmes. Other factors such as an increase in the number of community-based nutrition programs, increased socio-economic mobility and improved sanitation have all synergistically contributed to reduced malnutrition among Nepalese children.

Global goals focused on further improving the nutritional health of children have been established by the Sustainable Development Goals. These target the reduction of stunting by at least 40 % by 2025, wasting to be no higher than 5 % in 2025 and no increase in children with overweight/obesity^(4,5)^. Nepal has achieved the targeted reduction in stunting by reducing its prevalence from 57 % in 2001 to 25 % in 2022^(6)^. However, additional efforts are needed for Nepal to meet the Sustainable Development Goals for wasting and overweight. The prevalence of wasting has decreased from 11 % in 2001 to 8 % in 2022 and the proportion of children who were overweight increased from 0·7 % in 2001 to 1 % in 2022^(6)^. Thus, the sustained presence of wasting classifies nutritional status among children a high public health priority based upon standards set forth by the WHO^(7)^. At the same time, the increase in overnutrition places excess nutrition as an emerging public health issue. Continued progress will likely require more sophisticated approaches focused on the prevention and management of both undernutrition and overnutrition. To accomplish this, healthcare centres will need dedicated highly skilled and well-trained nutritionists/dieticians.

Kanti Children’s Hospital (KCH) is the only government paediatric hospital in Nepal. In 2020–2021, nearly 60 000 children and adolescents from different territories of Nepal visited the outpatient department and an additional 21 086 received emergency services^(8)^. KCH is among the top three institutions in Nepal providing care to children diagnosed with severe acute malnutrition; however, precise figures are unavailable as national health surveys obtain data at the community level and may not reflect the hospital setting^(1,6)^.

In 2018, KCH established a collaboration with the International Initiative for Pediatrics and Nutrition, located at Columbia University Irving Medical Center (New York), with the shared objective of advancing the provision and quality of nutritional care and conducting high-quality research in childhood nutrition^(9)^. International Initiative for Pediatrics and Nutrition provides clinician education, clinical infrastructure and mentorship in research to the clinical staff. Prior to the establishment of an expanded nutrition service within the Department of Pediatrics at KCH, it is essential to determine the prevalence of malnutrition, the variety and severity of nutritional conditions and predictors of poor nutrition status so that clinical research programmes may be efficiently designed and implemented. To this end, we report on the nutritional health and predictors of nutritional status in children and adolescents visiting Kanti Children’s Hospital.

## Materials and methods

Utilising a cross-sectional design for this pilot study, we recruited children and adolescents aged 0–15 years visiting either the inpatient or outpatient setting at KCH in Kathmandu, Nepal, during a 1-month period (April 2022). After written consent was obtained in the parent’s primary language, nutritional anthropometrics were assessed, and socio-demographic questionnaires were administered. Clinical data (e.g. reason for admission or visit to outpatient clinic) were abstracted from the medical chart. The study was approved by KCH’s Ethical Review Committee (reference number 963).

### Socio-demographics

Three questionnaires were utilised to collect information on socio-demographics. To ensure standardisation in the administration of the instruments and ensure representation from illiterate parents/caregivers, questions were read to study participants by the administrants. The WHO’s World Health Survey (2002) collected information on demographic and socio-economic characteristics, including parental education, occupation and self-reported ethnic group^(10)^. The Demographic and Health Wealth Index collected information on the family’s economic status as per previously published methodology^(11)^. The Demographic and Health consists of questions related to the household’s ownership of assets, such as a refrigerator or bicycle. The Food Insecurity Experience Scale questionnaire collected information on experiences and behaviours related to food access either due to lack of money or other resources, reflecting different levels of food insecurity^(12)^. The Food Insecurity Experience Scale questionnaire is composed of eight dichotomous (yes or no) questions related to food access over the preceding 12 months. Responses were aggregated with scores ranging from 0 to 8 and classified into three categories based on the global standard: food secure (0 to 3), moderately food insecure (4 to 6) and severely food insecure (7 to 8)^(12)^.

### Nutritional assessment

Anthropometric data were obtained and recorded by a study investigator. Height was measured to the nearest 0·1 cm using a portable stadiometer for children, and weight was measured to the nearest 0·05 kg using a calibrated digital scale. BMI and height *Z* scores for age and sex were classified based on the WHO standard growth charts^(13)^. A child with a height-for-age *Z* score of <-2 was classified as stunted^(14)^. A BMI-for-age *Z* score <-2 indicated wasting and >+2 indicated overweight^(14,15)^.

Mid-upper arm circumference (MUAC) was measured to the nearest 1 millimeter (mm) using a non-stretch tape around the midpoint between the olecranon process of the ulna and the acromial process of the scapula. For children 6 to 59 months old, a value <125 mm indicated wasting^(15)^. For children 5 years and older, MUAC was classified by *Z* score with a *Z* score of <-2 indicating wasting^(16)^. All groups of patients were classified by *Z* score for regression analysis using WHO reference standards^(17)^ for children less than 5 years old and Mramba *et al*.^(16)^ reference standards for children 5 years and older.

Head circumference was measured to the nearest 1 mm for all children under 5 years old using a non-stretch tape at the broadest part of the forehead, above the eyebrow and the ears and at the most prominent part of the back of the head. Classification was based on WHO growth reference standards where a *Z* score <-2 indicated microcephaly^(17)^.

### Statistical analysis

Demographic, clinical and nutritional data were input into a REDCap database and summarised using frequencies and proportions for categorical variables. For continuous variables with a normal distribution, mean and SD were reported, and for categorical values, number of patients (*n*) and percentages were reported. Individual wealth indices were derived utilising a multistep process that has been previously described^(18)^. Briefly, the dichotomous responses (yes or no) for patients’ assets (electricity, radio, etc.) were transformed into zero if negative or one if positive. Principal component analysis was then performed to obtain the relative contribution of each asset in the model^(11,19)^. The asset with the largest contribution in differentiating the variance in households’ wealth was considered the first component (PC1). The PC1 value was then multiplied by one for ‘yes’ responses and zero for ‘no’ responses resulting in a summary of the values. Results were then divided in tertiles, and individual scores were classified as Group 1 for scores ≤25^th^ percentile (e.g. lowest wealth), Group 2 for scores >25^th^ to <75^th^ percentile and Group 3 for scores ≥75^th^ percentile (e.g. highest wealth).

For comparison between groups, *χ* ^2^ test and multivariate binary logistic regression were used for categorical outcomes (e.g. stunting or healthy height). To investigate whether demographic and socio-economic status were prognostic indicators of nutritional status, groups were divided by age (under 5 years old and 5 years old and older) to align with standard reporting of nutritional indicators in children^(20,21)^. To determine variables related to the outcome of interest, a stepwise sensitivity analysis was performed. Variables with a significant association (*P* < 0·05) in a univariate model were included in the multivariate binary logistic regression model, with resulting variables yielding OR and 95 % CI. Statistical significance was set at *P* < 0·05. Data were analysed using Statistical Package for the Social Sciences (SPSS, version 23.0) and to perform the principal component analysis, PAST4 Software Package for Education and Data Analysis. WHO Anthro (version 2.0) and AnthroPlus (Geneva, Switzerland) software were used to graph nutritional indicators.

## Results

**Fig. 1:**
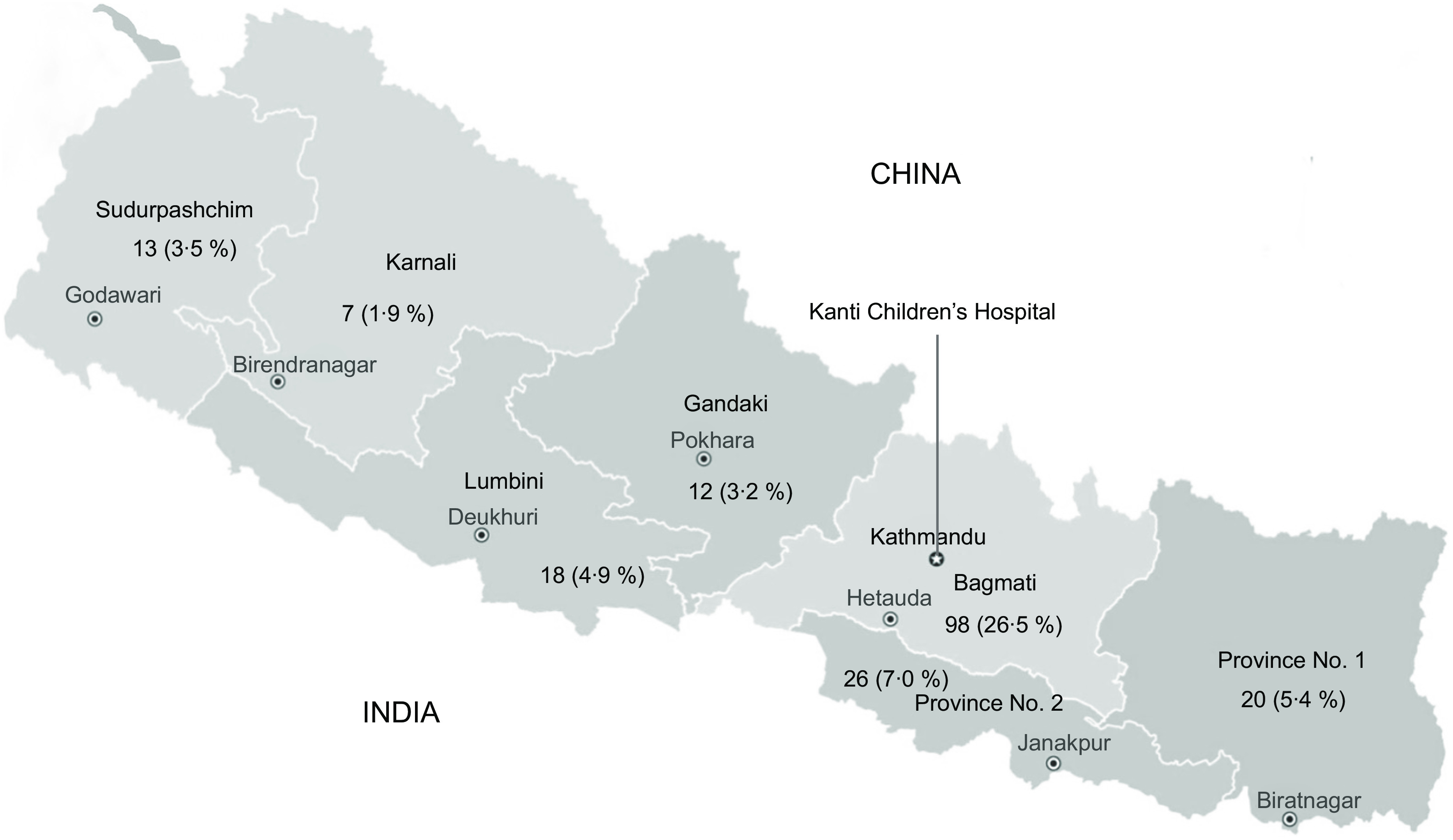
Geographical distribution of participants (*n* (%))

Our cross-sectional sample consisted of 370 participants. Similar to regional data^(22,23)^, 65·1 % were males and represented families from several provinces across Nepal (Table 1, Fig. 1). The mean age of the children was 3·9 years (sd ± 3·4 years). Children were recruited from the outpatient (*n* 165) and inpatient (*n* 205) settings. More than half of the participants (*n* 208) were being treated for an acute condition, while 43·8 % (*n* 162) were undergoing treatment for a chronic medical condition. The most common acute conditions included fever and pneumonia, while the most common chronic conditions were oncologic/haematologic (*n* 21), neurologic (*n* 15) and nephrology (*n* 10). Almost one-third of families (30·3 %) reported moderate to severe food insecurity and 41·6 % of participants reported affiliation with the Brahmin/Chhetri ethnicity, followed by the Janajati ethnicity (37·4 %) (Table 2).

**Table 1:** Participant and clinical characteristics

Variable	*n*	%	sd
Sex			
Male	241	65·1	
< 5 years old	170	64·4	
> 5 years old	71	67·0	
Female	129	34·9	
< 5 years old	94	35·6	
> 5 years old	35	33·0	
Age (years)			
All ages	370		
Mean ± sd	3·9		3·4
Minimum–Maximum (Range)	0·0–15·4		15·4
< 5 years old	264	71·6	
Mean ± sd	2·1		1·4
Minimum–Maximum (Range)	0·0–4·8		4·8
> 5 years old	106	28·4	
Mean ± sd	8·3		2·8
Minimum–Maximum (Range)	5·1–15·4		10·3
Hospital area			
Inpatient	205	55·4	
Outpatient	165	44·6	
Condition			
Acute	208	56·2	
< 5 years old	165	62·5	
> 5 years old	43	40·6	
Chronic	162	43·8	
< 5 years old	99	37·5	
> 5 years old	63	59·4	
Ward			
Emergency	4	1·1	
General OPD	131	35·4	
Medical	48	12·9	
Neurology/oncology/urology OPD	34	9·2	
Observation	55	14·9	
Oncology	22	5·9	
PICU	10	2·7	
Private ward	36	9·8	
Surgical	30	8·1	

OPD, outpatient department; PICU, paediatric intensive care unit.

**Table 2:** Socio-demographic characteristics

Characteristic	*n*	%
Respondent	369*	
Mother	241	65·1
Father	104	28·1
Primary caregiver other than parent	11	3·0
Other	13	3·5
Highest level of education	369	
No formal schooling	54	14·6
Less than primary school	33	8·9
Primary school completed	85	23·0
Secondary school completed	87	23·5
High school (or equivalent) completed	70	18·9
College/pre-university/University completed	23	6·2
Post graduate degree completed	17	4·6
Ethnic group	369	
Brahmin/Chhetri	154	41·6
Janajati	138	37·3
Dalit	41	11·1
Madhesi	36	9·7
Current job	369	
Government employee	18	4·9
Non-government employee	57	15·4
Self-employed	72	19·5
Employer	6	1·6
Not applicable, not working for pay	216	58·5
Wealth index	369	
Lowest wealth, Group 1 (≤ P25)	83	22·5
Middle wealth, Group 2 (> P25 to < P75)	194	52·6
Highest wealth, Group 3 (≥ P75)	92	24·9
Food insecurity	370	
Food secure	258	69·7
Moderately food insecure	63	17·0
Severely food insecure	49	13·3

* One patient did not answer the socio-demographic questionnaire.

### Nutritional indices

The prevalence of children classified as stunted was 25·9 % (Table 3). The prevalence of stunting was higher among children under 5 years of age (29·2 %) compared with children and adolescents 5 years of age and older (17·9 %; *P* = 0·026).

**Table 3:**
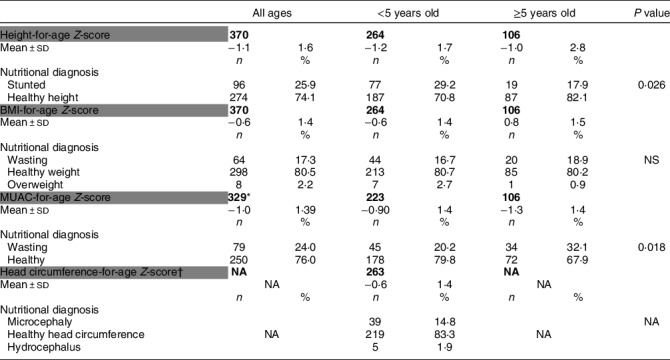
Distribution of nutritional assessment of the study participants

MUAC, mid-upper arm circumference; NS, not significant; NA, not applicable.

*Forty children under the age of 6 months old; 1 child with no data who is under the age of 4.

†Head circumference for age: Only applicable to children <5 years.

For children under 5 years of age, 16·7 % were classified with wasting according to BMI, whereas 20·2 % were classified with wasting according to MUAC. For children 5 years of age and older, 18·9 % were classified with wasting by BMI, and 32·1 % were classified with wasting by MUAC. A small percentage (2·2 %) of children and adolescents were classified as overweight. No significant differences in excess weight were observed by age groups. The prevalence of microcephaly was 14·8 % and was more prevalent among the Madhesi and Dalit groups with 14·3 % and 40 % (*P* = 0·002) of children diagnosed with microcephaly, respectively.

**Fig. 2:**
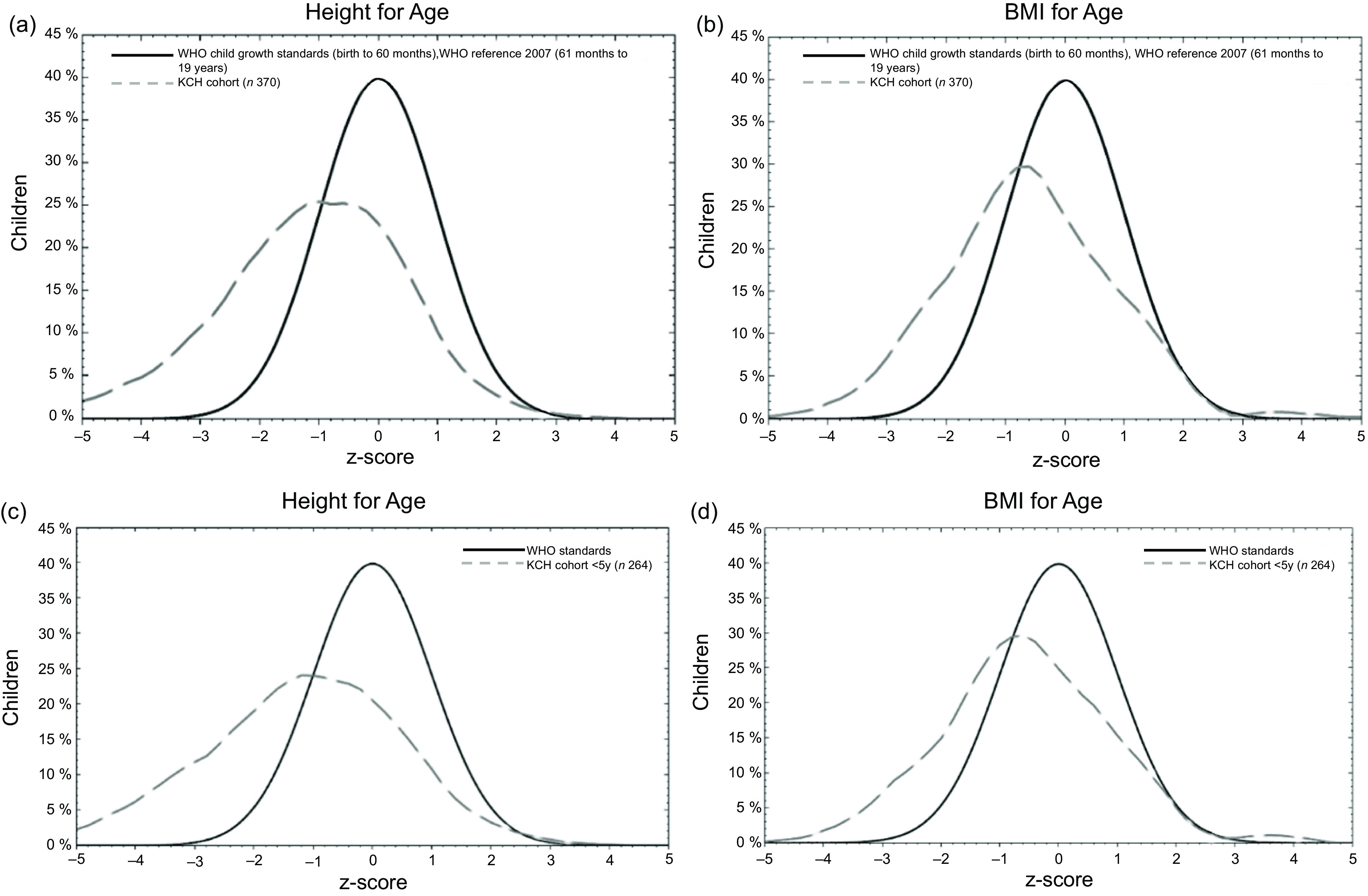
(a-d) Comparison of participants with the WHO child growth reference

To understand the growth of Nepalese children in relation to the WHO growth charts, *Z* score distributions from our dataset were compared with the WHO reference values (Fig. 2(a–d)). For both height- and BMI-for-age *Z* scores, the mean *Z* scores for Nepalese children were below the reference indicators. For both indicators, there was a left shift of the growth curve suggesting that Nepalese children are not developing as per global standards set forth by WHO. We then analysed growth curves by socio-demographic factors. Family self-reported ethnicity revealed poor growth was particularly evident for the Madhesi ethnicity followed by the Dalit ethnicity (see online Supplemental Fig. 1). For all ethnicities, height-for-age *Z* scores were below global reference values.

### Predictors of nutritional status

Univariate analysis revealed a significant association with nutritional status and medical condition (acute *v*. chronic), ethnicity, wealth index and education; however, no association was observed with food insecurity (see online Supplemental Table 1). Logistic regression analysis was performed to estimate the OR for stunting, wasting (defined by BMI-for-age and MUAC-for-age *Z* scores) and food insecurity (Table 4).

**Table 4:**
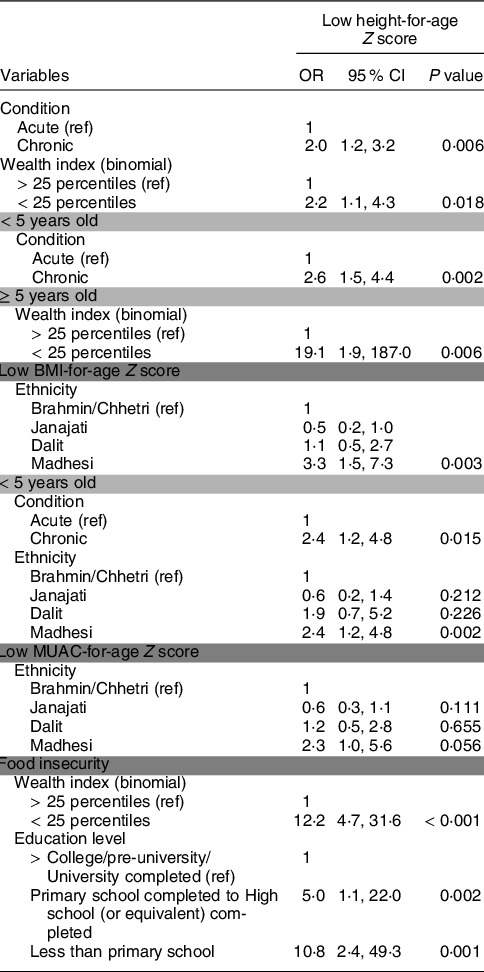
Predictors of nutritional status and food insecurity*

MUAC, mid-upper arm circumference.

* Variables with a significant association (*P* < 0·05) in the univariate model as predictors of nutritional status and food insecurity were included in the multivariate binary logistic regression model.

Children and adolescents with chronic medical conditions experienced a 2·0 increased odds (95 % CI (1·2, 3·2), *P* = 0·006) of stunting compared with children and adolescents with acute medical conditions. For children under the age of five, this figure increases to 2·6 (95 % CI (1·5, 4·4), *P* = 0·002). For wealth, families classified in ≤25^th^ percentile experienced a 2·2 increased odds (95 % CI (1·1, 4·3), *P* = 0·018) of stunting compared with the reference group (>25^th^ percentile). This was especially apparent for older children who experienced a 19·1 increased odds (95 % CI (1·9, 187·0), *P* = 0·006) of stunting. Families from the Madhesi ethnic group experienced a 3·3 increased odds (95 % CI (1·5, 7·3), *P* = 0·003) of wasting by BMI-for-age compared with the reference value. Children and adolescents from the Madhesi ethnic group also experienced a trend towards an increased odds of low MUAC-for-age *Z* score (OR 2·3, 95 % CI (1·0, 4·8), *P* = 0·056).

Contrary to the existing literature, there was not an association between nutritional status and food insecurity (see online Supplemental Table 2)^(24)^. Logistic regression revealed that children and adolescents in the lowest wealth index category (≤25 percentile) experienced 12·2 increased odds (95 % CI (4·7, 31·6), *P* < 0·001) of food insecurity compared with the reference group (>25 percentile). Education was also predictive of food insecurity as children from parents with less than primary school education experienced 10·8 (CI (2·4, 49·3), *P* = 0·001) increased odds of being food insecure as compared with the reference group.

## Discussion

Nepal is a low-income country that has achieved significant milestones in advancing children’s health through country-wide supplementation programmes, nutrition programmes and vaccine dissemination. Despite these advances, the prevalence of undernutrition remains high and factors predisposing children to poor nutrition in urban medical centres remain understudied. Our study begins to close this gap in knowledge by investigating the socio-demographic factors that may preemptively identify children who are at elevated risk of poor nutrition. The results from our study may be used to direct resource allocation so that timely and preemptive interventions may reverse or prevent adverse nutritional conditions from developing altogether.

The prevalence of stunting in our sample was 25·9 %. Stunting was higher among younger children compared with older children. Our data are higher than regional data (22 %)^(6)^ and another cross-sectional, hospital-based study (19 %)^(25)^. This may be reflective economic effects of COVID-19 particularly related to the rising cost of food and supply chain restrictions, but may also be reflective of the socio-demographic characteristics of the children served by this hospital. KCH is a government-supported hospital that provides care for families from a wide range of socio-economic backgrounds and is a preferred facility for the treatment of children from lower socio-economic families. Further, KCH is a regional referral centre for children with the most serious conditions. Our study found that children from the lowest wealth index were at risk for stunting, which is aligned with the findings of other studies in Nepal^(26,27)^. Children with a chronic condition were at risk for stunting, which may be due to continuous challenges with dietary intake due to the chronic disease itself or alterations in nutrient metabolism as a result of the medical condition. Similar to studies utilising national data^(26)^, we found that older age was predictive of stunting, which may be due to a limited number of nutrition rehabilitation programmes in previous years. Children from select ethnicities were at higher risk of stunting and Nepalese children tended to be shorter than norms set by the WHO, possibly due to malnutrition or genetics. Taken together, our data warrant additional research and underscores the importance of routine collection of height for all age groups as systematic monitoring leads to the prevention of stunting from developing altogether among younger age children^(28,29)^. As with previous successfully implemented nutrition programmes in Nepal^(4)^, our data suggest that national, multisector initiatives are needed to further achieve reductions in stunting among children and adolescence.

Compared with national data and other hospital-based surveys, we found a higher prevalence of wasting among our sample (8 % and 9·2 % (on admission) *v*. 17·3 %, respectively)^(6,25)^. Several factors may account for this discrepancy. First, the children in our study represent children suffering from either an acute or chronic medical condition, both of which can cause wasting. Additionally, KCH serves as a national referral hospital especially for children with severe disease, refractory conditions or rare diseases. Thus, the children served by KCH may be especially vulnerable to wasting due to the complexity of their condition. Additionally, the majority of children were from middle to low socio-economic families. Similar to our finding among children with stunting, ethnicity was also predictive of wasting. We also found that growth differed by ethnicity as illustrated from comparisons to the WHO growth standards. Despite these variations, our data confirm that wasting remains alarmingly high, as defined by global standards^(7)^, among Nepalese children in the hospital setting and comprehensive nutritional programmes fostering the prompt delivery of nutritional care are urgently needed.

Of notable concern, we observed a high prevalence of stunting and wasting among children 5 years of age and older. Global age limitations in access to nutritional rehabilitation products and national nutritional policies focused on children under 5 years of age may be contributing factors. The repercussions of undernutrition in children beyond the age of five are not inconsequential as poor body image, reduced work and learning capacity and poor reproductive health in adolescence have been reported^(30,31)^. Unfortunately, existing policies continue to focus on children under 5 years of age and adolescent girls^(32,33)^. Our findings suggest a re-evaluation of current nutritional programmes to ensure adequate provision of nutritional care for older children.

Our results align with previous data in that the use of BMI alone in children and adolescents is not a comprehensive indicator of nutritional status^(16,34)^. There are well-established limitations of the use of BMI alone for classifying nutritional status, and several research groups have underscored the importance, ease of use and low cost of utilising MUAC in the paediatric setting^(34,35)^. Our study found that the classification of wasting using BMI-for-age z-score and MUAC among children under the age of five was inconsistent. These results were expected and further highlight the importance of including assessment of height, weight and MUAC in paediatric nutritional assessments^(16,34)^.

Our data revealed that the percentage of children classified as overweight is higher in our study compared with national data, 2·7 % compared with 1 %, respectively^(6)^. However, these figures must be considered recognising that the majority of our population resides in an urban setting. While these numbers remain relatively low compared with surrounding countries, our data suggest that overweight is increasing among Nepalese children and could emerge as a public health crisis in the upcoming years. Importantly, our data advocate for Health Ministries, clinicians and clinical investigators to establish public health programmes targeting the prevention of obesity with the objective of attenuating the rise in overweight status, an objective aligned with the Sustainable Development Goals.

The prevalence of microcephaly was 14·8 %, which is lower than previously reported (24–56 %)^(36,37)^. Head circumference has been shown to vary by ethnic group with it most commonly observed among the Dalit ethnicity^(38)^, which we also observed. The lower prevalence may be due to the small representation among our participants. National data suggest that Dalits have consistently poor performance in regard to neonatal and childhood mortality, maternal health service utilisation, childhood nutritional status, wealth index and education^(39,40)^. Other studies have found that microcephaly is associated with stunting and lower socio-economic status^(41)^. The association between microcephaly and socio-economic status may be explained by the high presence of maternal undernutrition in impoverished communities contributing to prenatal nutritional deprivation^(41)^. Taken together, specific programmes that reach out to the most underserved and economically disadvantaged groups are needed to close the remaining gaps in maternal and childhood undernutrition in Nepal.

Our study identified several risk factors for undernutrition. Contrary to our expectations, food insecurity, wealth and education were not consistently associated with all nutritional indicators, perhaps due to children in our study already seeking medical care. Our insignificant findings may also be related to the small sample size and limited representation of ‘at-risk’ groups. We found that the prevalence of wasting was high among the Madhesi ethnicity, which has been reported in other studies in Nepal^(42)^. This was an expected finding given that national surveys have demonstrated differences among ethnic groups in terms of health service utilisation and health outcomes in Nepal, where underprivileged groups, such as the Madhesi group, fare far worse than more privileged groups^(39)^.

Aligned with previous studies^(43–45)^, we found that a large percentage of children with wasting also report moderate to severe food insecurity (60·9 %) despite the results not reaching statistical significance. Our findings may be explained by the majority of families reporting food security or the reallocation of resources to young children when food is scarce^(45)^. Studies have shown that households reallocate food to prioritise feeding young children during periods of food insecurity^(46)^. Other factors may have a stronger effect on malnutrition such as substantial improvements in access to health and nutrition services, household wealth and parental education^(4)^. The association between parental education and nutritional status was only significant when assessed by MUAC, perhaps due to MUAC being a more sensitive indicator of wasting, especially in the setting of disease^(47,48)^. Parental education has been associated with wasting in other studies in Nepal and elsewhere^(49,50)^, as mothers who are less educated have been shown to have the poorest child feeding practices^(4)^. With this finding, focused interventions aimed at strengthening maternal education may be drawn.

Our results must be interpreted in light of several limitations. First, this was a single-institution study located in the populous Bagmati province and may not be reflective of other national hospitals within or outside of this province. Our study collected data over a single month and variations in nutritional status over time (e.g. summer *v*. winter months) was not obtained. Due to personnel limitations, several outpatient clinics were not included in the study including the psychiatric department, burn unit and neonatal intensive care unit. Children from the inpatient setting were recruited at any point during their hospital admission; therefore, duration of hospital stay was also not collected. Finally, we were limited in our analyses due to the relatively small sample size. Our pilot study aimed to establish an initial snapshot of nutritional morbidities in the largest, government-supported children’s hospital in the country and was not intended to establish country-wide indicators on the nutritional health of children in Nepal. Future research, within the context of a clinical research program, is in development at KCH to examine these nutritional conditions prospectively.

Hospital-based nutrition programmes improve the nutritional status of children^(51)^. However, challenges with adequate resources, particularly in countries with a large youth such as Nepal, is a challenge for the medical field until capacity and skills are further cultivated to meet the need. As KCH and International Initiative for Pediatrics and Nutrition advance their collaboration, several recommendations may be drawn from our study to guide the development of our collaborative hospital-based nutrition programme. First, utilising both MUAC and BMI-for-age to identify wasting is essential due to the limitations of BMI-for-age. Nutritional programmes need to be designed to meet the needs of children of all ages and likely prioritise children with chronic medical conditions as these often require consistent parental education and ongoing monitoring. Nutrition education may be a low-cost intervention for disadvantaged families and may aid in the prevention of all forms of malnutrition^(52)^. Finally, collaboration and referral to community-based nutrition programmes is likely essential to sustain and build upon progress obtained in the hospital.

## Supporting information

Chapagain et al. supplementary material Chapagain et al. supplementary material

## Supplementary material

For supplementary material accompanying this paper visit https://doi.org/10.1017/S136898002300188X
