# Supplementary material for: A cross-sectional study evaluating the prevalence and predictors of malnutrition among children and adolescents visiting an urban academic hospital in Nepal
Source: Public Health Nutr. 2023 Oct 9;26(12):2738–47. doi: 10.1017/S136898002300188X (PMC10755450; doi:10.1017/S136898002300188X)
Supplement: Chapagain et al. supplementary material [file S136898002300188Xsup001.docx]

**Supplementary Figure 1.** Comparison of Nepal Ethnic groups with World Health Organization child growth standards/reference

| **Ethnic group** | **Height-for-Age** | **BMI-for-Age** | **MUAC-for-Age** |
| --- | --- | --- | --- |
| Brahmin/Chhetri (All children) | 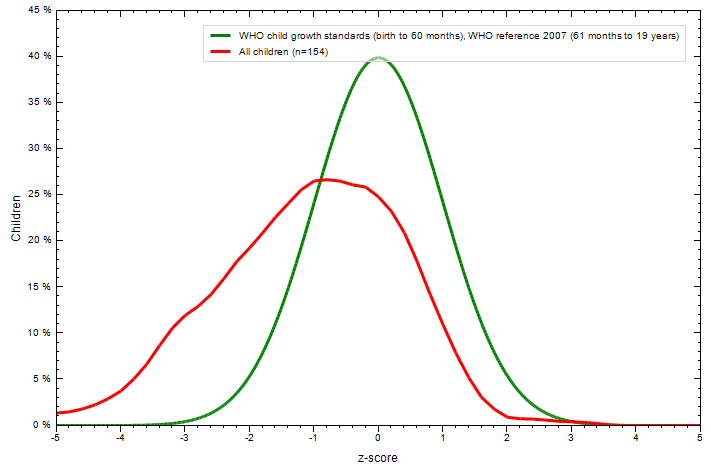 | 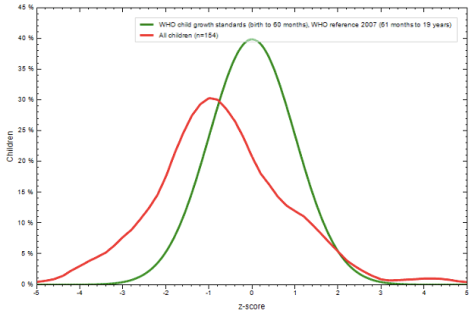 | NA |
| < 5y | 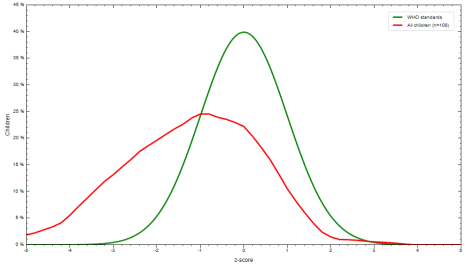 | 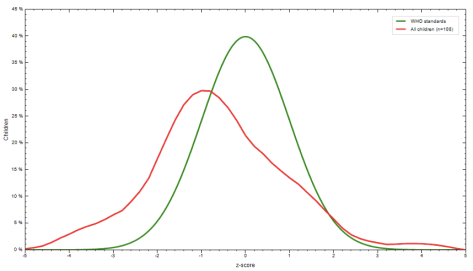 | 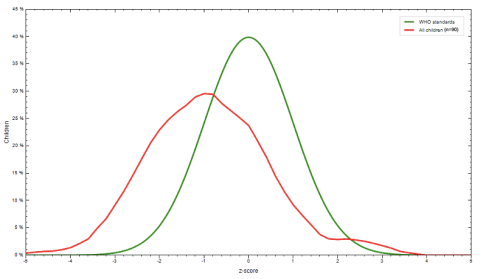 |
| Janajati  (All children) | 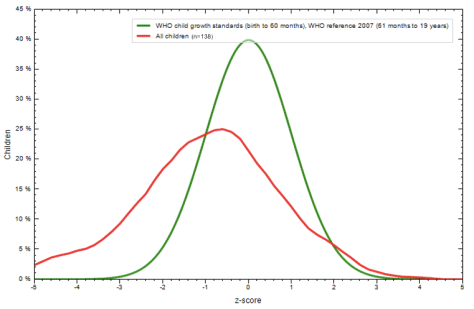 | 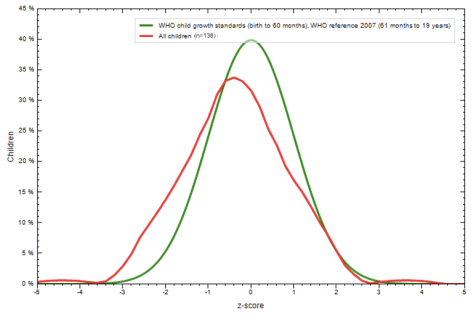 | NA |
| < 5y | 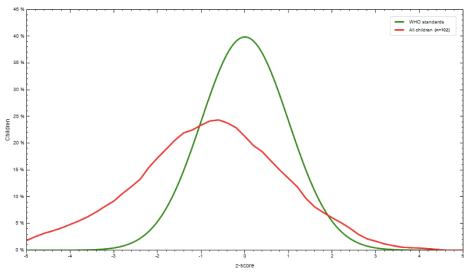 | 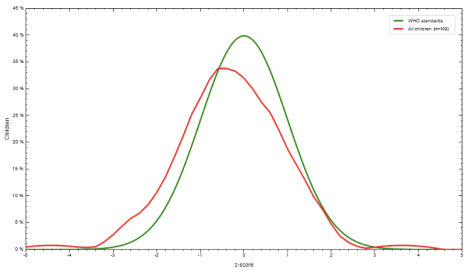 | 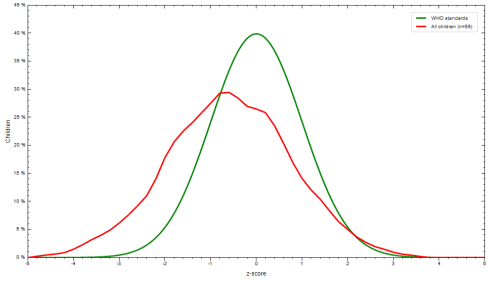 |
| Dalit  (All children) | 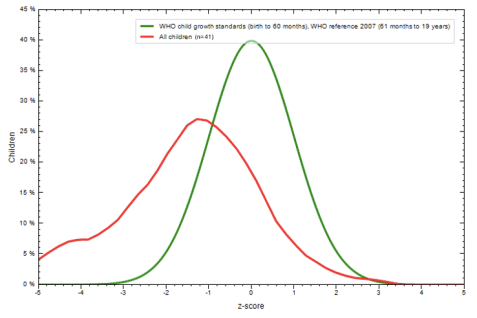 | 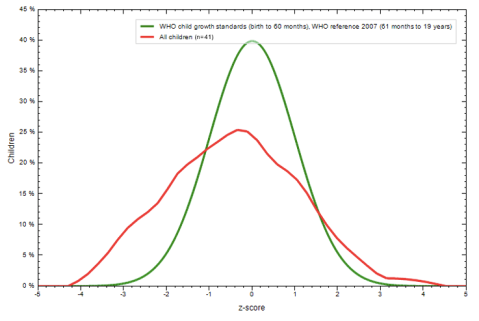 | NA |
| < 5y | 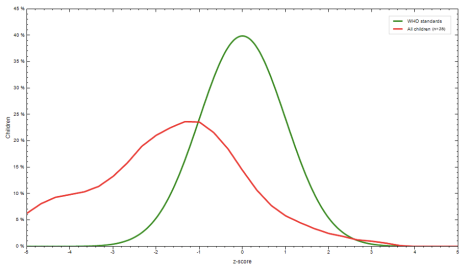 | 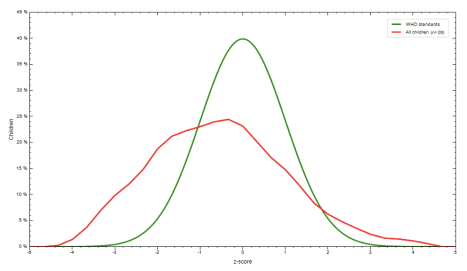 | 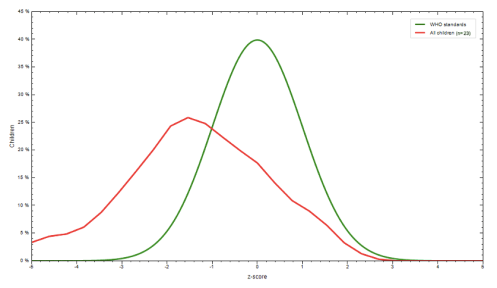 |
| Madhesi  (All children) | 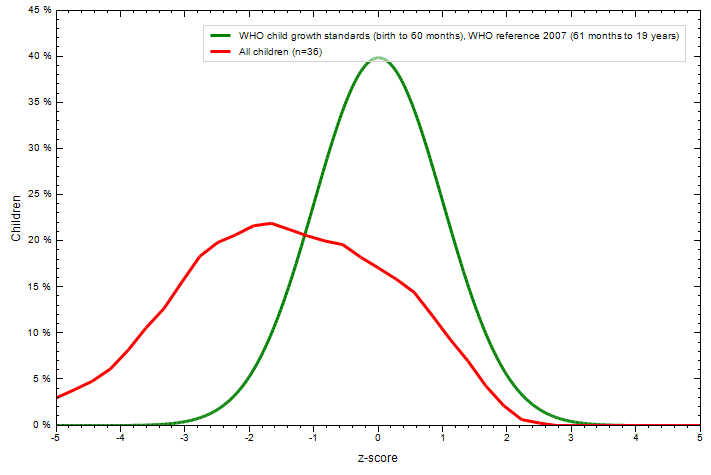 | 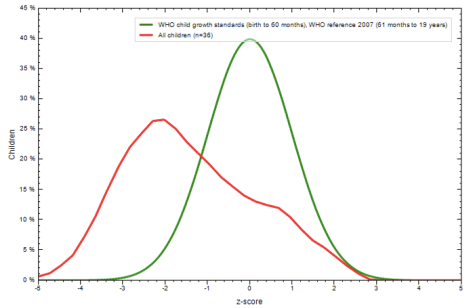 | NA |
| < 5y | 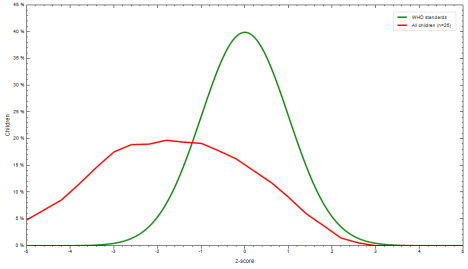 | 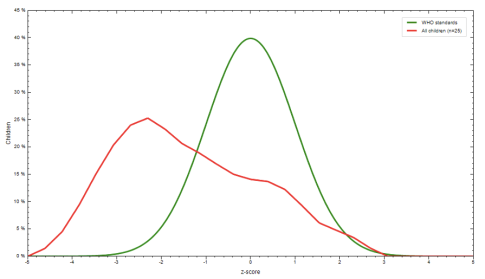 | 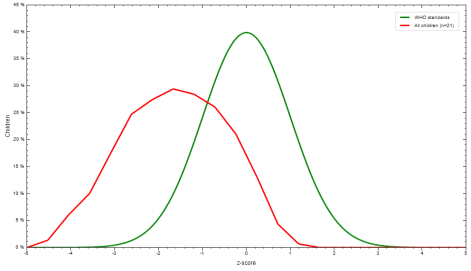 |

Abbreviations: BMI, body mass index; MUAC, mid-upper arm circumference; NA, not applicable.

**Supplementary Table 1**. Univariate Analysis of Nutritional Status and Socioeconomical Indicators

|  | **Height-for-age Z-Score** | | | **BMI-for-Age Z-Score** | | | **MUAC-for-Age Z-Score** | | |
| --- | --- | --- | --- | --- | --- | --- | --- | --- | --- |
|  | **Healthy**  n (%) | **Stunted**  n (%) | **P value** | **Healthy/OW**  n (%) | **Wasting**  n (%) | **P value** | **Healthy**  n (%) | **Wasting**  n (%) | **P value** |
| **Ward**  Outpatient  Inpatient | 145 (75.9)  129 (72.1) | 46 (24.1)  50 (27.9) | NS | 165 (86.4)  141 (78.8) | 26 (13.6)  38 (21.2) | NS | 138 (81.7)  112 (70.0) | 31 (18.3)  48 (30.0) | 0.041 |
| **Condition**  Acute  Chronic | 167 (80.3)  107 (66.0) | 41 (19.7)  55 (34.0) | 0.008 | 182 (87.5)  124 (76.5) | 26 (12.5)  38 (23.5) | 0.022 | 147 (81.7)  103 (69.1) | 33 (18.3)  46 (30.9) | 0.019 |
| **Province**  No.1  Bagmati  Madhesh  Sudur-Paschim  Lumbini  Karnali  Gandaki | 14 (70.0)  72 (73.5)  19 (73.1)  10 (76.9)  13 (72.2)  5 (71.4)  7 (58.3) | 6 (30.0)  26 (26.5)  7 (26.9)  3 (23.1)  5 (27.8)  2 (28.6)  5 (41.7) | NS | 15 (75.0)  82 (83.7)  18 (69.2)  10 (76.9)  12 (66.7)  6 (85.7)  10 (83.3) | 5 (25.0)  16 (16.3)  8 (30.8)  3 (23.1)  6 (33.3)  1 (14.3)  2 (16.7) | NS | 16 (84.2)  65 (78.3)  14 (56.0)  7 (58.3)  9 (56.3)  3 (60.0)  7 (58.3) | 3 (15.8)  18 (21.7)  11 (44.0)  5 (41.7)  7 (43.8)  2 (40.0)  5 (41.7) | NS |
| **Ethnicity**  Brahmin/Chhetri  Janajati  Dalit  Madhesi | 117 (76.0)  106 (76.8)  28 (68.3)  23 (63.9) | 37 (24.0)  32 (23.2)  13 (31.7)  13 (36.1) | NS | 127 (82.5)  125 (90.6)  33 (80.5)  20 (55.6) | 27 (17.5)  13 (9.4)  8 (19.5)  16 (44.4) | <0.001 | 104 (76.5)  102 (82.9)  27 (73.0)  16 (50.0) | 32 (23.5)  21 (17.1)  10 (27.0)  16 (50.0) | 0.002 |
| **Wealth Index^a^**  Group 1 (<p25)  Group 2  Group 3 (>p75) | 50 (60.2)  151 (77.8)  73 (79.3) | 33 (39.8)  43 (22.2)  19 (20.7) | 0.020 | 67 (80.7)  164 (84.6)  74 (80.4) | 16 (19.3)  30 (15.4)  18 (19.6) | NS | 44 (62.0)  142 (80.7)  63 (77.8) | 27 (38.0)  34 (19.3)  18 (22.2) | 0.003 |
| **Education Level^b^**  < LTPS  PSC to HSC  > CC | 58 (66.7)  183 (75.6)  33 (82.5) | 29 (33.3)  59 (24.4)  7 (17.5) | NS | 73 (83.9)  196 (81.0)  36 (90.0) | 14 (16.1)  46 (19.0)  4 (10.0) | NS | 52 (65.0)  169 (79.7)  28 (77.8) | 28 (35.0)  43 (20.3)  8 (22.2) | 0.029 |
| **Food Insecurity**  Food Secure  Moderate FI  Severe FI | 195 (75.6)  43 (68.3)  36 (73.5) | 63 (24.4)  20 (31.7)  13 (26.5) | NS | 214 (82.9)  48 (76.2)  44 (89.8) | 44 (17.1)  15 (23.8)  5 (10.2) | NS | 177 (78.7)  41 (70.7)  32 (69.6) | 48 (21.3)  17 (29.3)  14 (30.4) | NS |

Abbreviations: BMI, body mass index; MUAC, mid-upper arm circumference; OW, overweight; NS, not significant; LTPS, less than primary school completed; PSC, primary school completed; HSC, high school completed; CC, college completed; FI, food insecure.

^a^Wealth Index (3 categories): Group 1 = <p25, Group 2 = > p25, Group 3 = ≥p75.

^b^Education level (3 categories): ≤ Less than primary school, Primary school completed to High school (or equivalent) completed, ≥ College/pre-university/University completed.

**Supplementary Table 2.** Food Insecurity Distribution as a Secondary Nutritional Parameter

|  | **Food Insecurity**  **(All participants)** | | | | **Food Insecurity**  **(< 5 y)** | | | | **Food Insecurity**  **(≥ 5 y)** | | | |
| --- | --- | --- | --- | --- | --- | --- | --- | --- | --- | --- | --- | --- |
|  | Food Secure  n (%) | Moderate Food Insecurity  n (%) | Severe Food Insecurity  n (%) | **P value** | Food Secure  n (%) | Moderate Food Insecurity  n (%) | Severe Food Insecurity  n (%) | **P value** | Food Secure  n (%) | Moderate Food Insecurity  n (%) | Severe Food Insecurity  n (%) | **P value** |
| **Ward**  Outpatient  Inpatient | 145 (75.9)  113 (63.1) | 20 (10.5)  43 (24.0) | 26 (13.6)  23 (12.8) | 0.003 | 115 (78.8)  73 (61.9) | 15 (10.3)  30 (25.4) | 16 (11.0)  15 (12.7) | 0.003 | 30 (66.7)  40 (65.6) | 5 (11.1)  13 (21.3) | 10 (22.2)  8 (13.1) | NS |
| **Condition**  Acute  Chronic | 148 (71.2)  110 (67.9) | 31 (14.9)  32 (19.8) | 29 (13.9)  20 (12.3) | NS | 123 (74.5)  65 (65.7) | 21 (12.7)  24 (24.2) | 21 (12.7)  10 (10.1) | NS | 25 (58.1)  45 (71.4) | 10 (23.3)  8 (12.7) | 8 (18.6)  10 (15.9) | NS |
| **Province**  No.1  Bagmati  Madhesh  Sudur-Paschim  Lumbini  Karnali  Gandaki | 8 (40.0)  68 (69.4)  11 (42.3)  10 (76.9)  14 (77.8)  2 (28.6)  10 (83.3) | 8 (40.0)  16 (16.3)  9 (34.6)  3 (23.1)  4 (22.2)  3 (42.9)  2 (16.7) | 4 (20.0)  14 (14.3)  6 (23.1)  0 (0.0)  0 (0.0)  2 (28.6)  0 (0.0) | 0.021 | 4 (40.0)  52 (73.2)  5 (29.4)  4 (57.1)  9 (69.2)  1 (25.0)  7 (77.8) | 4 (40.0)  10 (14.1)  7 (41.2)  3 (42.9)  4 (30.8)  2 (50.0)  2 (22.2) | 2 (20.0)  9 (12.7)  5 (29.4)  0 (0.0)  0 (0.0)  1 (25.0)  0 (0.0) | 0.028 | 4 (40.0)  16 (59.3)  6 (66.7)  6 (100.0)  5 (100.0)  1 (33.3)  3 (100.0) | 4 (40.0)  6 (22.2)  2 (22.2)  0 (0.0)  0 (0.0)  1 (33.3)  0 (0.0) | 2 (20.0)  5 (18.5)  1 (11.0)  0 (0.0)  0 (0.0)  1 (33.3)  0 (0.0) | NS |
| **Ethnicity**  Brahmin/Chhetri  Janajati  Dalit  Madhesi | 122 (79.3)  89 (64.5)  25 (61.0)  21 (58.3) | 17 (11.0)  28 (20.3)  9 (22.0)  9 (25.0) | 15 (9.7)  21 (15.2)  7 (17.0)  6 (16.7) | NS | 90 (83.3)  65 (63.7)  18 (64.3)  14 (56.0) | 9 (8.3)  22 (21.6)  8 (28.6)  6 (24.0) | 9 (8.3)  15 (14.7)  2 (7.1)  5 (20.0) | 0.010 | 32 (69.6)  24 (66.7)  7 (53.8)  7 (63.6) | 8 (17.4)  6 (16.7)  1 (7.7)  3 (27.3) | 6 (13.0)  6 (16.7)  5 (38.5)  1 (9.1) | NS |
| **Wealth Index^a^**  Group 1 (<p25)  Group 2  Group 3 (>p75) | 37 (44.6)  134 (69.1)  86 (93.5) | 24 (28.9)  35 (18.0)  4 (4.3) | 22 (26.5)  25 (12.9)  2 (2.2) | < 0.001 | 26 (44.8)  97 (70.3)  64 (95.5) | 18 (31.0)  25 (18.1)  2 (3.0) | 14 (24.1)  16 (11.6)  1 (1.5) | < 0.001 | 11 (44.0)  37 (66.1)  22 (88.0) | 6 (24.0)  10 (17.9)  2 (8.0) | 8 (32.0)  9 (16.1)  1 (4.0) | 0.022 |
| **Education Level^b^**  ≤ LTPS  PSC to HSC  ≥ CC | 41 (47.1)  178 (73.6)  38 (95.0) | 22 (25.3)  41 (16.9)  0 (0.0) | 24 (27.6)  23 (9.5)  2 (5.0) | < 0.001 | 24 (50.0)  138 (73.4)  25 (92.6) | 11 (22.9)  34 (18.1)  0 (0.0) | 13 (27.1)  16 (8.5)  2 (7.4) | < 0.001 | 17 (43.6)  40 (74.1)  13 (100.0) | 11 (28.2)  7 (13.0)  0 (0.0) | 11 (28.2)  7 (13.0)  0 (0.0) | < 0.001 |

Abbreviations: NS, not significant; LTPS, less than primary school completed; PSC, primary school completed; HSC, high school completed; CC, college completed.

^a^Wealth Index (3 categories): Group 1 = <p25, Group 2 = > p25, Group 3 = ≥p75.

^b^Education level (3 categories): ≤ Less than primary school, Primary school completed to High school (or equivalent) completed, ≥ College/pre-university/University completed.
